# Efficacy of Lifestyle Interventions in Reducing Weight and BMI Among People With Type 2 Diabetes: A Six-Month Clinical Trial

**DOI:** 10.7759/cureus.92153

**Published:** 2025-09-12

**Authors:** Shweta Malakar, Shivendra Kumar Singh, Kauser Usman

**Affiliations:** 1 Department of Community Medicine, King George's Medical University, Lucknow, IND; 2 Department of Community Medicine and Public Health, King George's Medical University, Lucknow, IND; 3 Department of Medicine, King George's Medical University, Lucknow, IND

**Keywords:** bmi management, lifestyle intervention, lifestyle modification counselling, randomized clinical trial, types 2 diabetes, weight and bmi, weight reduction

## Abstract

Background

Type 2 diabetes mellitus (T2DM) is often associated with overweight and obesity, contributing to poor glycemic control and increased risk of complications. Lifestyle modification is a cornerstone in the management of T2DM, but its real-world impact on anthropometric measures like weight and body mass index (BMI) requires further evaluation.

Objective

The objective of the study was to assess the effectiveness of lifestyle modification counseling on weight and BMI reduction among people with T2DM over a six-month period.

Methods

This randomized controlled trial included 110 participants with T2DM, equally divided into intervention (n=55) and control (n=55) groups. The intervention group received structured lifestyle counseling, including dietary advice, physical activity recommendations, and behavioral strategies, while the control group received routine clinical care. Anthropometric parameters (weight and BMI) were recorded at baseline and at the six-month follow-up. Statistical analysis was performed using paired and unpaired t-tests with significance set at p<0.05.

Results

At baseline, there were no significant differences between groups in weight, BMI, or waist-hip ratio (p>0.05), confirming comparability. At six months, the intervention group showed a significant mean weight reduction of 2.04 ± 2.30 kg (p<0.001) and BMI reduction of 0.792 ± 1.009 kg/m² (p<0.001). The control group showed non-significant changes in both parameters. Between-group comparisons revealed a significantly greater weight reduction in the intervention group (p=0.001), although the BMI difference was not statistically significant (p=0.924).

Conclusion

Structured lifestyle modification counseling significantly improved weight and BMI in people with T2DM over six months, highlighting its role as an effective, non-pharmacological strategy for obesity management in diabetes care.

## Introduction

Type 2 diabetes mellitus (T2DM) is a progressive metabolic disorder characterized by insulin resistance, β-cell dysfunction, and chronic hyperglycemia. It accounts for approximately 90-95% of all diabetes cases globally and poses a significant public health challenge [1]. According to the International Diabetes Federation (IDF) Diabetes Atlas 2021, 537 million adults aged 20-79 years were living with diabetes worldwide, with projections reaching 643 million by 2030 and 783 million by 2045 [2]. India, with over 77 million individuals affected, ranks second globally in diabetes prevalence, often referred to as the "diabetes capital" of the world [3].

Obesity and overweight, quantified primarily through body mass index (BMI) and body weight, are key modifiable risk factors in the development and progression of T2DM. Studies suggest that individuals with a BMI ≥25 kg/m² have a three to seven-fold increased risk of developing T2DM compared to those with a normal BMI [4]. Furthermore, weight reduction of as little as 5-7% of baseline body weight has been shown to significantly improve glycemic control, insulin sensitivity, and reduce the risk of diabetes-related complications [5].

Lifestyle interventions comprising dietary modifications, increased physical activity, and behavioral counseling form the cornerstone of non-pharmacological management of T2DM [6,7]. Multiple large-scale trials, such as the Diabetes Prevention Program (DPP) in the United States and the Finnish Diabetes Prevention Study, have demonstrated the effectiveness of lifestyle changes in reducing the incidence of T2DM by 58% in high-risk individuals [8]. In the Indian context, the Indian Diabetes Prevention Programme (IDPP-1) showed that lifestyle modification alone reduced the progression to diabetes by 28.5% in individuals with impaired glucose tolerance [9].

Despite these established benefits, implementation of structured, periodic lifestyle counseling in routine clinical care remains limited, especially in low- and middle-income countries. Moreover, there is a paucity of short-term, real-world clinical trials that assess the anthropometric benefits of such interventions among patients already diagnosed with T2DM.

This study aims to evaluate the efficacy of structured lifestyle intervention, including diet and physical activity counseling, in reducing weight and BMI over a six-month period among patients with T2DM. The objective is to generate clinical evidence on the effectiveness of non-pharmacological strategies in improving anthropometric outcomes, which may serve as a vital component in comprehensive diabetes management.

## Materials and methods

Study design and setting

This was a randomized controlled trial (RCT) conducted over a six-month period to evaluate the effect of lifestyle modification counseling on weight and BMI in people with T2DM. The study was conducted at the Medicine Outpatient Department (OPD) of King George’s Medical University (KGMU), Lucknow, Uttar Pradesh, India.

Ethical Considerations

Prior approval was obtained from the Institutional Ethics Committee of KGMU (approval number: 905/Ethics/18, reference code: 93rd ECM II B-Ph.D./P2). Written informed consent was taken from all participants after explaining the purpose and procedure of the study. All data were anonymized to ensure confidentiality. This trial was registered in Clinical Trials Registry-India (registration number: CTRI/2020/07/026780).

Study population

The study population consisted of adult patients diagnosed with T2DM attending the Medicine OPD. The inclusion and exclusion criteria for the study population are given below.

Inclusion Criteria

The study population included patients diagnosed with T2DM who were on oral hypoglycemic agents, aged 30-65 years, and willing to participate and provide informed consent.

Exclusion Criteria

Patients with T2DM who were on insulin therapy, pregnant or lactating women, individuals with severe physical disabilities or comorbidities that could restrict physical activity, and patients unwilling or unable to comply with follow-up visits were excluded.

Sample size calculation 

The sample size was calculated using a formula appropriate for comparing means between two independent groups, based on assumed population variance (σ²) and expected mean difference (d) as follows:

$n = \frac{(Z_{\alpha} + Z_{\beta})^{2} \cdot 2\sigma^{2}}{d^{2}}$

where Z_α_ is the critical value of the normal distribution at α one tailed (e.g. for a confidence level of 95%, α is 0.05 one tailed and the critical value is 1.645), Z_β_ is the critical value of the normal distribution at β (e.g. for a power of 80%, β is 0.2 and the critical value is 0.84), σ^2^ is the population variance, d is the difference to be detect. Incidence or prevalence rates were not used in this calculation. Thus, putting all the values in the formula,

$n = \frac{(1.645 + 0.84)^{2} \cdot 2 \cdot (1)^{2}}{(0.5)^{2}} = 49.4018 \approx 50$

The sample size was thus calculated to be 50 in each group (control and experimental). So, the total sample size including both groups was 100. However, for managing dropouts and non-cooperation from study subjects, we included 10% more than the total sample size, and thus, the number of subjects enrolled in the study was estimated to be 110, i.e., 55 in each group. Ultimately, the number of patients enrolled in the control group was 58, and in the intervention group, it was 54. After loss to follow-up, there were 52 patients in each group. The ratio of the two study groups, experimental and control, was 1:1. 

Randomization, blinding, and group allocation

**Figure 1:**
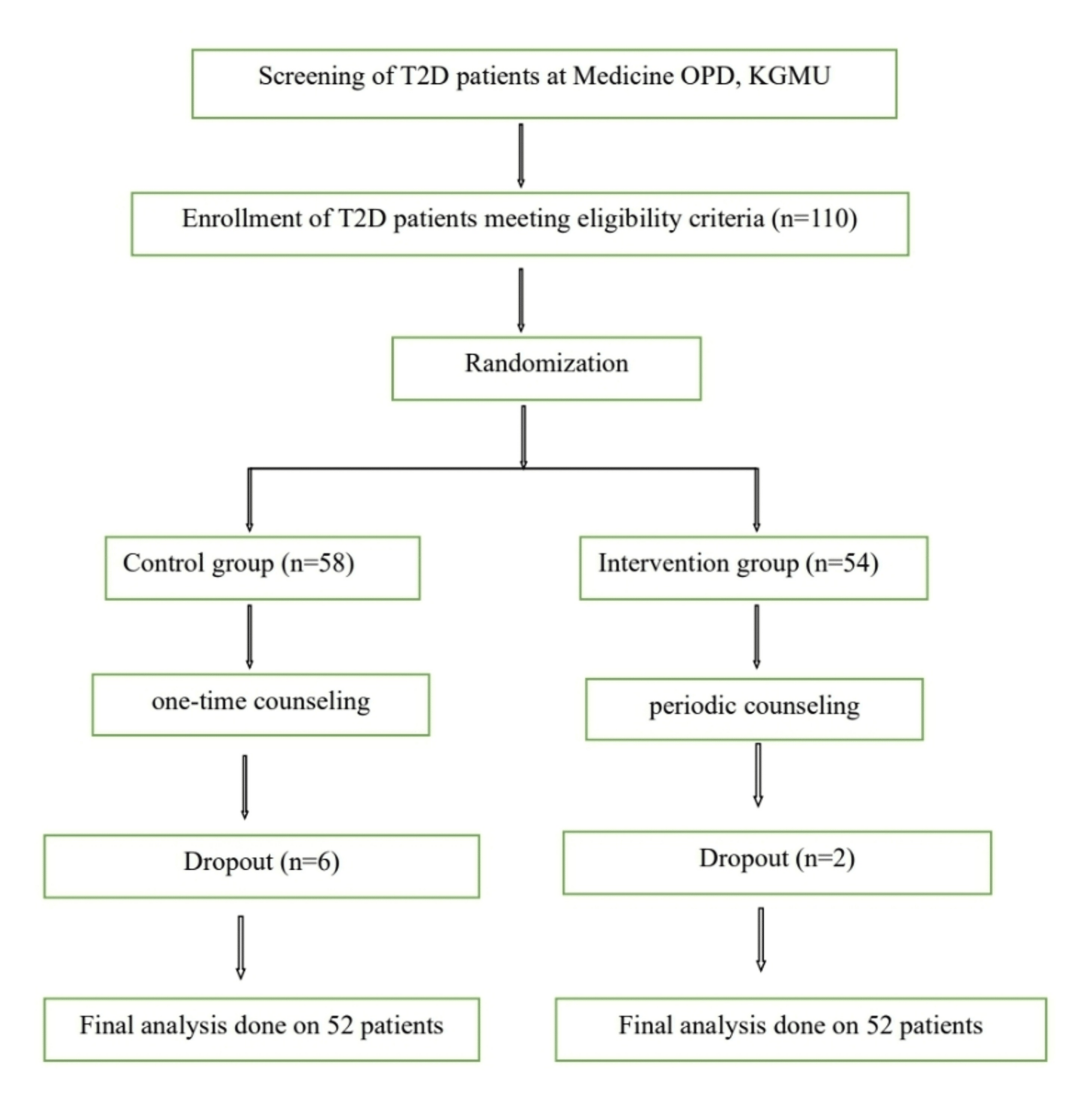
CONSORT diagram CONSORT: Consolidated Standards of Reporting Trials; KGMU: King George's Medical University; T2DM: type 2 diabetes mellitus

Participants were randomly assigned to either the control or experimental group using a computer-generated randomization sequence. The study was single-blinded; the study participants were blinded, i.e., unaware of the intervention group. Allocation concealment was ensured to minimize selection bias. The Consolidated Standards of Reporting Trials (CONSORT) guidelines were followed, and the CONSORT diagram is given in Figure 1.

Intervention

Control Group

The participants in the control group received only standard advice about dietary modifications, physical activity, and healthy lifestyle choices (one-time lifestyle modification counseling) for diabetes management during a routine OPD visit. This one-time counselling session was of approximately 30-45 minutes and was conducted in person.

Intervention Group

The participants in the intervention group received structured lifestyle modification counseling once a month for six months. These sessions included: nutritional education focused on balanced meals and portion control, a personalized diet chart, and physical activity recommendations tailored to individual capabilities, motivation, and behavioral tips to improve adherence to lifestyle changes. Each counseling session lasted approximately 30-45 minutes and was conducted in person, with telephonic reminders and follow-up support in between visits.

Outcome measures

The primary outcomes assessed were change in weight (in kg) and change in BMI (in kg/m²). These measurements were recorded at baseline and again at the end of the six-month intervention period using standard equipment and procedures.

Data collection and analysis

Weight and BMI were recorded for all participants at baseline and after six months using standardized procedures and calibrated equipment. Data were collected in a pre-designed format and entered into Microsoft Excel (Microsoft Corporation, Redmond, Washington, United States) for organization. Statistical analysis was performed using IBM SPSS Statistics for Windows, version 24.0 (Released 2016; IBM Corp., Armonk, New York, United States). Descriptive statistics such as mean and standard deviation (SD) were used to summarize baseline characteristics. Paired t-tests were applied to assess within-group differences before and after the intervention, while independent t-tests were used to compare outcomes between the control and experimental groups at the end of the study. A p-value of less than 0.05 was considered statistically significant.

## Results

Baseline characteristics of study participants

Table 1 presents the baseline anthropometric measurements and duration of diabetes for both the control and intervention groups. The mean weight, BMI, and waist-hip ratio were comparable between the two groups, with no statistically significant differences. There was no significant difference between the control and intervention groups in weight, BMI, and WHR at baseline, ensuring comparability of the groups.

**Table 1:** Baseline anthropometry and duration of diabetes Independent t-tests for continuous variables; p-value less than 0.05 is significant

Variable	Control Group (n=52), mean±SD	Intervention Group (n=52), mean±SD	95% CI	p-value
Weight (kg)	66.67 ± 11.51	67.14 ± 11.19	-4.96 to 4.01	0.84
BMI (kg/m²)	25.61 ± 4.09	26.29 ± 6.01	-2.71 to 1.34	0.50
Waist-to-hip ratio	0.88 ± 0.04	0.89 ± 0.04	-0.03 to 0.00	0.14
Duration of diabetes (years)	3.39 ± 3.48	- 4.67 ±6.19	-3.25 to 0.69	0.20

Comparison of BMI at six-month follow-up

Table 2 compares the BMI after six months between the two groups. BMI reduction at six months was not significantly different between the two groups, indicating that while lifestyle interventions showed a positive trend, the overall difference was not statistically significant.

**Table 2:** BMI of the two groups at the six-month follow-up Independent t-test for continuous variable BMI (body mass index); p-value less than 0.05 is significant

Measurement	Control Group (n=22), mean±SD	Intervention Group (n=22), mean±SD	95% CI	p-value
BMI (Kg/m²) after 6 months	25.42 ± 3.79	25.51 ± 14.87	-1.91 to 1.73	0.92

Comparison of weight reduction from baseline to six-month follow-up

Table 3 illustrates the weight reduction in both groups over six months. The intervention group experienced a significantly greater reduction in weight (2.04 ± 2.30 kg) compared to the control group (0.47 ± 2.21 kg) (p-value<0.001). This suggests that lifestyle interventions had a positive impact on weight loss.

**Table 3:** Weight reduction at six months from baseline Independent t-tests for weight reduction (continuous variable) ^**^p-value less than 0.01 is highly significant

Group	Weight Reduction (kg), mean difference ±SD	95% CI		p-value
		Lower	Upper	
Control	0.47 ±2.21	-2.46	-0.68	0.001**
Intervention	2.04 ±2.30			

Weight reduction in the control and intervention groups

Table 4 presents weight reduction within the control group, which was not significant (p = 0.135). This indicates that without structured interventions, weight reduction was minimal.

**Table 4:** Weight reduction in the control group at six months from baseline Paired t-test for weight reduction (continuous variable); p-value less than 0.05 is significant

Group	Weight Reduction (kg), mean difference ± SD	t value	p-value	95% confidence interval	
				Lower	Upper
Control Group	0.47 ±2.21	1.519	0.135	-.152	1.09

Table 5 presents the within-group analysis of weight reduction in the intervention group, which showed a significant weight reduction (p < 0.0001). This confirms that lifestyle interventions were effective in promoting weight loss.

**Table 5:** Weight reduction in the intervention group at six months from baseline Paired t-test for weight reduction (continuous variable) ^**^ p-value less than 0.01 is highly significant

Group	Weight Reduction (Kg), mean difference ± SD	t value	p-value	95% Confidence Interval	
				Lower	Upper
Intervention Group	2.04 ±2.27	6.26	0.0001**	1.41	2.67

BMI reduction in the control and intervention groups

Table 6 illustrates the BMI reduction in the control group, which was not significant (p = 0.178), further highlighting the effectiveness of lifestyle interventions in the intervention group.

**Table 6:** BMI reduction in the control group after six months from baseline Paired t-test for continuous variable BMI (kg/m²) *: A p-value less than 0.05 is significant. **: A p-value less than 0.01 is highly significant

Group	BMI Reduction (kg/m^2^), mean difference ±SD	t value	p-value	95% confidence Interval	
				lower	Upper
Control Group	0.16 ±0.84	1.37	0.178	-.075	.39

Table 7 illustrates the BMI reduction in the intervention group over six months. A significant BMI reduction was observed in the intervention group (p < 0.0001), suggesting that lifestyle interventions contributed to BMI improvements.

**Table 7:** BMI reduction in the intervention group after six months from baseline Paired t-test for continuous variable BMI (kg/m^2^) ^**^ p-value less than 0.01 is highly significant

Group	BMI Reduction (kg/m^2^), mean difference ±SD	t value	p-value	95% Confidence Interval	
				Lower	Upper
Intervention Group	0.79 ±1.01	5.55	0.0001**	0.51	1.08

## Discussion

The present study evaluated the effect of lifestyle modification counseling on weight and BMI reduction among patients with T2DM over a six-month period. The within-group analysis revealed a statistically significant reduction in both weight (mean reduction of 2.04 ± 2.30 kg) and BMI in the intervention group, underscoring the efficacy of structured lifestyle interventions, although the between-group comparison for BMI at six months did not reach statistical significance. These findings contribute to the growing body of evidence supporting lifestyle interventions for T2DM management and align with numerous studies identified through a comprehensive review of the literature.

Our results are consistent with the Look AHEAD (Action for Health in Diabetes) clinical trial, which demonstrated that intensive lifestyle interventions lead to significant weight loss (approximately 8.6% of initial body weight) and BMI reduction in overweight or obese individuals with T2DM over one year [10]. The greater magnitude of weight loss in the Look AHEAD trial may be attributed to its longer duration and more intensive intervention, including frequent counseling sessions and meal replacements, compared to our six-month program with less intensive follow-up.

Similarly, the DPP funded by the National Institute of Diabetes and Digestive and Kidney Diseases achieved a mean weight loss of 5-7% over 2.8 years [11], which is higher than our observed 2.04 kg reduction. This difference may be due to the program's longer duration and structured goal-setting, including 150 minutes of weekly physical activity, which was not explicitly mandated in our study. Ma et al. adapted DPP lifestyle interventions among overweight or obese adults in their study and reported that lifestyle modification, targeting a 7% weight loss through diet and physical activity, significantly reduced the risk of T2DM progression in individuals with impaired glucose tolerance [12].

A meta-analysis by Chen et al. reported an average weight reduction of 2-2.7 kg over six months across multiple RCTs [13], closely mirroring our findings. Another meta-analysis by Huang et al. found that lifestyle interventions, including dietary counseling and exercise, resulted in a mean BMI reduction of 0.8-1.2 kg/m² over 6-12 months [14], supporting the clinical relevance of our observed BMI improvements. These meta-analyses highlight the consistency of modest but meaningful anthropometric improvements with lifestyle interventions, particularly in short-term studies like ours.

The China Da Qing Diabetes Prevention Outcome Study by Gong et al. reported significant BMI improvements (approximately 1.07 kg/m² reduction) in the intervention group over six years through diet and exercise [15]. While their study’s longer duration allowed for greater cumulative effects, our study’s results suggest that even shorter interventions can achieve comparable early benefits, particularly in resource-limited settings.

An Indian study by Ramachandran et al. on urban Indian subjects with impaired glucose tolerance found that structured lifestyle modification significantly reduced BMI and body weight, with a mean weight loss of 1.5-2 kg over three years [9]. This aligns closely with our findings and underscores the applicability of lifestyle interventions in Indian populations, where dietary patterns and cultural factors may differ from Western cohorts.

In contrast, a study by Wing et al. on the long-term effects of lifestyle interventions in T2DM patients reported significant initial weight loss (approximately 6.0 kg at one year) but noted that sustained BMI reductions required ongoing support and reinforcement beyond the initial intervention period [16]. A systematic review by Norris et al. found that diabetes self-management education, including lifestyle counseling, significantly reduced body weight (mean reduction of 1.7-2.5 kg) when programs exceeded six months [17]. Another RCT by Wadden et al. reported that behavioral counseling combined with nutritional advice led to a 3.5% weight loss and significant BMI improvements in T2DM patients over 12 months [18]. These findings support our observation that structured, personalized counseling is critical for anthropometric improvements. It also highlights a limitation of our six-month study, as the lack of long-term follow-up may limit the sustainability of our observed outcomes. 

Additional studies further support our findings. A systematic review by Terranova et al. found that lifestyle-based weight loss interventions in T2DM patients resulted in a mean weight loss of 3.2 kg at 12 months, though interventions achieving less than 5% weight loss did not consistently improve metabolic outcomes [19]. This suggests that our 2.04 kg weight loss, while clinically meaningful, may need to be sustained or increased to optimize metabolic benefits. Benasi et al. evaluated a sequential intervention combining well-being therapy and lifestyle intervention in T2DM patients, reporting significant weight reductions at the six-month follow-up [20], similar to our findings. The inclusion of psychological components in their intervention highlights the potential for integrating behavioral strategies to enhance outcomes. A systematic review by Galaviz et al. of global diabetes prevention interventions found a mean weight loss of 2.3 kg across 22 translational DPPs, with variability due to intervention dose and delivery agent [21]. Our study’s results fall within this range, reinforcing the feasibility of modest interventions in diverse settings. A study by Debussche et al. on quarterly outpatient lifestyle counseling in T2DM patients reported a 2.5 kg weight loss at six months, reinforcing the efficacy of regular counseling [22]. The structured follow-up in this study aligns with our approach, though our intervention was less frequent.

A meta-analysis by Pillay et al. corroborated that lifestyle interventions incorporating dietary advice and physical activity significantly improved BMI and weight in T2DM patients, with greater effects in programs with frequent contact points and culturally tailored content [23]. A Finnish diabetes prevention study by Tuomilehto et al. reported that lifestyle intervention achieved a 4.2% weight loss at one year, significantly improving insulin sensitivity in participants with impaired glucose tolerance [8]. Similarly, a meta-analysis by Franz et al. reviewed nutrition practice guidelines for T2DM, finding that medical nutrition therapy led to a 2-3 kg weight loss over 6-12 months [24]. Our study’s focus on personalized counseling and the emphasis on dietary counseling aligns with these findings, suggesting that patient-centered approaches enhance intervention efficacy.

An RCT by Wang et al. tested a behavioral lifestyle intervention with mobile and connected tools, finding that T2DM patients who self-monitored their progress lost more weight (approximately 3.5 kg) in the first three months compared to controls [25]. A recent RCT by St-Jules et al. evaluated technology-supported counseling in T2DM patients with chronic kidney disease, reporting a 3.5% weight loss at three months, suggesting that technology-enhanced interventions may amplify short-term outcomes [26]. This supports the role of technology in enhancing adherence, which could be a future direction for similar interventions. These findings indicate the potential for digital tools to sustain results.

Finally, a recent systematic review by Sarker et al. of lifestyle interventions in low- and middle-income countries found significant reductions in weight (approximately 2-3 kg) and BMI, particularly in Southeast Asian settings [27]. This supports the applicability of our findings in resource-limited contexts.

Collectively, our findings are consistent with a robust body of evidence supporting the effectiveness of structured lifestyle interventions in reducing weight and BMI among T2DM patients. However, variations in study duration, intervention intensity, follow-up mechanisms, and cultural factors account for differences in the magnitude and sustainability of results. Studies with longer durations [10,15] report greater cumulative effects, while our six-month intervention demonstrates that even modest, resource-efficient programs can yield clinically meaningful outcomes in resource-limited settings. Future research should explore strategies to sustain these benefits, such as integrating digital health tools or community-based support systems.

Strengths and limitations of the study

The RCT design minimized bias and enhanced the reliability of the results of this study. The focused lifestyle counseling was tailored and consistent, improving adherence. The follow-up period of six months allowed sufficient time to assess intervention impact on the weight and BMI of the study population.* *However, there were also some limitations*. *The small sample size of this study may limit generalizability. The short duration of this study prevents assessment of the long-term sustainability of lifestyle changes. The self-reported adherence to lifestyle modifications could introduce recall bias.

Future directions

Future studies should aim for a larger, more diverse population and a longer follow-up period to assess the long-term sustainability and effectiveness of lifestyle modifications. Additionally, integrating technology-based interventions like mobile health apps or tele-counseling may enhance adherence and scalability. Exploring the psychosocial and behavioral determinants of lifestyle adherence could also guide personalized intervention strategies.

## Conclusions

This study demonstrates that structured lifestyle modification counseling is effective in significantly reducing weight and BMI among patients with T2DM over a six-month period. While between-group differences in BMI were not statistically significant, the intervention group showed a marked within-group improvement in both weight and BMI, highlighting the positive impact of personalized dietary guidance, physical activity promotion, and behavioral support. These findings reinforce the importance of incorporating lifestyle intervention programs into routine diabetes management to achieve meaningful anthropometric improvements and potentially better long-term metabolic outcomes.
